# Inferior Vena Cava Tumor Thrombus in the Emergency Department: A Case Report

**DOI:** 10.5811/cpcem.38065

**Published:** 2025-03-20

**Authors:** Victor Cisneros, Leila Danishgar, Nisan Verma, Ami Kurzweil

**Affiliations:** * Eisenhower Health, Department of Emergency Medicine, Rancho Mirage, California; † University of Queensland, Ochsner Clinical School, New Orleans, Louisiana; ‡ University of California, Irvine, Department of Emergency Medicine, Orange, California

**Keywords:** inferior vena cava, tumor thrombus, congestive heart failure, thromboembolism

## Abstract

**Introduction:**

The inferior vena cava (IVC) drains a significant portion of the lower body. Pathologies associated with the IVC can present significant diagnostic and therapeutic challenges. We present a case of IVC tumor thrombus in the emergency department.

**Case Report:**

A 76-year-old male with symptoms of volume overload was evaluated, leading to the diagnosis of IVC mass likely from tumor thrombus.

**Conclusion:**

Patients with volume overload should be evaluated for both heart failure and presence of a potential thrombus. Point-of-care ultrasound and other imaging modalities play crucial roles in early diagnosis. Prompt identification and differentiation between bland and tumor thrombi are vital for appropriate management, potentially improving patient outcomes.

## INTRODUCTION

The inferior vena cava (IVC) is the largest vein in the body, responsible for draining a significant portion of the lower body.^1^ Pathologies associated with the IVC, including thrombosis and neoplasms, can present significant diagnostic and therapeutic challenges, especially when first identified in the emergency department (ED).^2^

Inferior vena cava thrombosis is a rare process that usually stems from a congenital abnormality, but it is found even more rarely from an acquired cause.^2^ The most common acquired cause of IVC thrombosis is from an unretrieved IVC filter.^2^ Other than this, patients with a combination of a predisposing hypercoagulable state and prothrombotic conditions and/or abdominal pathologies are at risk for IVC thrombosis as well.^2^,^3^ For these reasons, IVC abnormalities should be assessed carefully to guide clinical-decision making and improve patient care.

Acquired causes of IVC thrombus can be further stratified into bland thrombus, pseudo-thrombus, and primary and secondary malignancies.^1^ A tumor thrombus is the presence of a tumor extending into blood vessel walls such as the portal vein or the IVC.^4^ Malignancies like this can have intravascular extensions in underlying leiomyosarcoma and renal cell carcinoma. Differentiation between a bland thrombus and tumor thrombus is vital for determining therapeutic approach and can be done with ultrasound, magnetic resonance imaging (MRI), and computed tomography (CT).^5^ In the ED, ultrasound has revolutionized point-of-care imaging, providing faster diagnoses, especially in thrombus identification.^5^ Treatments vary greatly depending on clinical identification of the thrombus but may include anticoagulation, chemotherapy, thrombolysis, and surgical resection.^6^ There is limited research available on the topic of IVC tumor thrombus, which warrants further research.

In this report, we discuss the case of a man who sought emergent care for worsening dyspnea and fatigue as well as noticeable leg swelling, secondary to IVC obstruction.

## CASE REPORT

A 76-year-old male with a history of atrial fibrillation, diabetes mellitus, and hepatitis presented to the emergency department with progressive shortness of breath and generalized weakness over five days. Physical examination revealed bilateral lower extremity edema, decreased breath sounds in the right lower lung field, 2+ bilateral pitting edema to mid-lower legs, and motor weakness with chronic, right-foot drop. Electrocardiogram showed atrial fibrillation with rapid ventricular response, and laboratory tests revealed normocytic anemia, hyperglycemia, and elevated blood urea nitrogen/creatinine levels. Troponin levels were elevated, with values of 382 nanograms per liter (ng/L) at two hours and 429 ng/L at four hours (reference range <40 ng/L). However, the B-type natriuretic peptide (BNP) was normal at 96 picograms per milliliter (pg/mL) (<100 pg/mL).

**Image 1:**
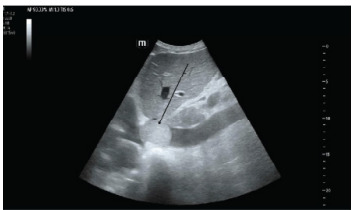
Point-of-care ultrasound of the inferior vena cava (IVC) showing a hyperechoic circular mass in the proximal IVC (arrow) adjacent to the right atrium. Distal IVC is seen plethoric.

**Image 2:**
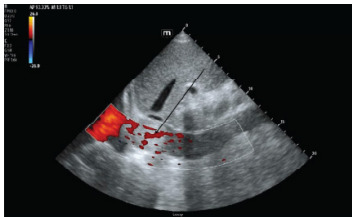
Point-of-care ultrasound of the inferior vena cava (IVC) with color Doppler applied showing poor venous return (arrow) to the right atrium secondary to IVC mass/tumor thrombosis.

A point-of-care cardiac ultrasound to evaluate for volume overload and cardiac function revealed a well-circumscribed mass in the IVC near the right atrium (Images 1 and 2). Chest radiograph showed mild pulmonary vascular congestion. A subsequent CT angiogram of the chest, abdomen, and pelvis demonstrated an ill-defined hypodense area at the intrahepatic and suprahepatic portions of the IVC, raising concern for a mass. Additional findings included mild hepatomegaly, a nodule in the left urinary bladder, and lymphadenopathy in the mediastinal, aortocaval, and periportal regions, suggesting neoplasm and metastases.

The on-call vascular surgeon recommended transfer to a higher level of care for oncological and surgical evaluation. However, the patient declined surgical intervention and opted for hospice care.

**CPC-EM Capsule**

What do we already know about this clinical entity?

*Inferior vena cava (IVC) obstruction can have multiple etiologies and lead to severe systemic consequences, thus crucially requiring an early and accurate diagnosis*.

What makes this presentation of disease reportable?

*A potential IVC tumor thrombus can present clinically similar to Congestive heart failure (CHF), making it easy to miss if not correctly identified early*.

What is the major learning point?

*Point-of-care ultrasound (POCUS) can be useful to quickly differentiate between IVC occlusions/tumor thrombus and typical CHF secondary to right-sided heart failure*.

How might this improve emergency medicine practice?

*Rapid differentiation of an IVC occlusion using POCUS can expedite diagnosis of IVC tumor thrombus and initiate proper treatment and management*.

## DISCUSSION

The impairment and/or obstruction of the IVC can lead to severe, systemic consequences, making early detection and accurate diagnosis crucial. Abnormalities of the IVC can stem from congenital malformations, trauma, or acquired diseases that result in compression and hinder venous return.^1^,^2^ Among the risk factors for acquired IVC-related illnesses are infection, obesity, vascular diseases, pregnancy, and malignancy.^3^,^7^,^8^ Tumor thrombus of the IVC, while rare, is a critical finding that can present with varying symptom severity depending on its size and location. The Mayo Clinic’s staging system for tumor thrombus classification, ranging from level 0 (thrombus extending into the renal vein) to level 4 (thrombus extending into the supradiaphragmatic IVC or the right atrium), provides a structured approach for assessment.^9^ The presented case, with a level 4 thrombus, underscores the complexity and severity of near-complete occlusion of the IVC neighboring the right atrium.^9^

Differentiating between a bland thrombus and a tumor thrombus is vital due to their differing treatment approaches. A bland thrombus carries the risk of embolization and dissemination, necessitating early identification to implement strategies such as IVC filters, anticoagulation, and thrombolytics.^5^ In contrast, surgical resection remains the primary curative approach for a tumor thrombus, as pharmacological interventions are typically ineffective and the risk of metastases is significant. Historically, conventional venography was the gold standard for diagnosing venous thrombosis. However, CT and MRI now provide reliable readings, with ultrasound, particularly color Doppler ultrasound, serving as a valuable first-line modality.^5^ Computed tomography is often used initially to identify IVC pathologies due to its effectiveness in detecting abdominal abnormalities. Notably, some studies have identified a “streak and thread” sign as indicative of tumor thrombus on CT.^10^ For visualizing an IVC tumor thrombus, certain types of MRI are considered more reliable than CT and have the advantage of not using ionizing radiation.^11^ Doppler ultrasound is beneficial in demonstrating abnormal or reduced blood flow caused by an IVC mass, although its effectiveness can be limited by operator experience and artifacts from adjacent structures.^11^,^12^

Emerging imaging modalities such as contrast-enhanced ultrasound (CEUS) offer a comprehensive view, as tumor thrombi typically include small vessels that can be distinctly visualized due to blood-pooling contrast enhancement.^13^ Both CEUS and Doppler ultrasound provide expedited, cost-effective imaging compared to MRI and CT, maintaining high sensitivity and specificity. This case aligns with these findings, as both Doppler ultrasound and CT demonstrated IVC occlusion.

Diagnosing an IVC tumor thrombus can be particularly challenging, as it can mimic the clinical presentation of congestive heart failure (CHF). This mimicry is due to shared symptoms and hemodynamic consequences. Congestive heart failure, both left-sided and right-sided, typically arises from the heart being unable to maintain adequate cardiac output. This leads to compensatory responses that, while initially adaptive, exacerbate fluid retention, vascular resistance, and myocardial remodeling, ultimately increasing systemic congestion and cardiac dysfunction. The patient in this case report, with a normal BNP of 96 pg/mL and no prior history of heart failure, presented similarly to a CHF patient. Both IVC thrombus and CHF may manifest with symptoms of systemic venous congestion, including dyspnea, orthopnea, and lower extremity edema.^1^–^3^ The obstruction of venous return by an IVC tumor thrombus can elevate venous pressures, leading to fluid extravasation into the interstitial space and increasing systemic venous pressure. Over time, if the left heart is unable to compensate for this increase in systemic venous pressure, there will be an elevation in pulmonary venous pressure. This will reduce lung compliance and impair gas exchange, resulting in dyspnea and subsequent pulmonary congestion. This mirrors the pathophysiological cascade of CHF. Additionally, an IVC tumor thrombus can exacerbate pre-existing cardiac dysfunction or predispose individuals to decompensated heart failure.

In the context of this patient, it may be valuable to compare the effects of an IVC tumor thrombus to those of CHF, particularly secondary to right-sided heart failure. As in CHF, obstruction of the IVC impairs venous return, leading to decreased right ventricular preload and subsequent overcompensation by the right heart. Over time, this compensatory mechanism may progress to contractile dysfunction, ultimately impairing the cardiac output of the right ventricle and resulting in right-sided heart failure, characterized by hepatomegaly, ascites, and peripheral edema, which can be indistinguishable from advanced CHF manifestations. Distinguishing between an IVC tumor thrombus and CHF is crucial for guiding appropriate therapeutic interventions.^14^

While diuretics and vasodilators are cornerstone treatments for CHF, managing an IVC mass may require interventions ranging from anticoagulation to surgical resection. Surgical options for IVC tumor thrombus may include the need for sternotomy, cardiopulmonary bypass, and coronary artery bypass graft, with the choice of surgery depending on the size and location of the thrombus.^15^ Swift and accurate recognition is essential for determining the appropriate surgical route.

Had the patient not elected hospice care, further evaluation of the symptoms and mass through advanced imaging and diagnostic modalities, such as echocardiography, chest CT, positron-emission tomography (PET) or biopsy, would have been essential. An echocardiogram could have provided an estimate of ejection fraction and offered detailed visualization of hemodynamic changes, enhancing the overall clinical assessment. A chest CT would have supported the findings of pulmonary congestion observed on the chest radiograph and provided a more comprehensive view, potentially identifying smaller or early-stage pathologies that might have been missed on the radiograph.^5^ Additionally, PET demonstrating vessel expansion and a biopsy confirming neoplastic cell distribution would have established a definitive diagnosis of tumor thrombus.^10^

The absence of these diagnostic steps represents a significant limitation of this case report. A further limitation is the absence of CEUS, a modality particularly effective in visualizing thrombosis. Although Doppler ultrasound was chosen for its speed and efficacy, CEUS could have provided additional comparative insights, enhancing the diagnostic accuracy. Despite these limitations, this case underscores the critical importance of recognizing and diagnosing IVC tumor thrombus to guide appropriate clinical management.

## CONCLUSION

Inferior vena cava tumor thrombosis can present with a wide range of symptoms and subsequently cause significant morbidity. This case underscores the pivotal role of ultrasound in the prompt identification of an IVC tumor thrombus, enabling timely and diagnostic therapeutic interventions. This rare case did not have a correspondingly obvious presentation; however, with thorough preliminary workup, a level four IVC tumor thrombus was identified.

Given the overlap in clinical presentation between IVC tumor thrombus and CHF secondary to right-sided heart failure, ultrasound (Doppler/CEUS) can be used as a rapid and cost-effective frontline imaging modality, allowing clinicians to tailor treatment plans and investigate accordingly. It is important to keep this pathology in mind as part of the differential diagnosis. Therefore, integrating ultrasound into the diagnostic algorithm for patients presenting with symptoms suggestive of venous congestion is imperative for timely and accurate diagnosis of IVC occlusion. With continued testing, the identification of IVC tumor thrombus can be confirmed, which gives better direction to proper treatments and outcomes.
